# New advances in sequence assembly

**DOI:** 10.1101/gr.223057.117

**Published:** 2017-05

**Authors:** Adam M. Phillippy

**Affiliations:** National Human Genome Research Institute, National Institutes of Health, Bethesda, Maryland 20892, USA

It may be hard to believe, but the idea of sequence assembly is around 40 years old. Consider this pair of quotes from Rodger Staden (Staden 1979):

“With modern fast sequencing techniques and suitable computer programs it is now possible to sequence whole genomes without the need of restriction maps.”

“If the 5′ end of the sequence from one gel reading is the same as the 3′ end of the sequence from another the data is said to overlap. If the overlap is of sufficient length to distinguish it from being a repeat in the sequence the two sequences must be contiguous. The data from the two gel readings can then be joined to form one longer continuous sequence.”

Replace “gel reading” with “read” and these sentences would go unnoticed in the introduction of any paper today. Here you can also see the birth of jargon that now pervades the field: *overlaps* between *reads* form *contigs* (contiguous sequences). Just a few months later, Gingeras et al. (1979) described “Computer programs for the assembly of DNA sequences.” It all sounds so modern, until the discussion mentions FORTRAN code stored on magnetic tapes.

How, then, can we fill an entire special issue of *Genome Research* with “new advances” so many years later? To me, this reflects the beauty of the problem—simple enough to be stated in a single paragraph, yet complex enough to sustain a field of research for decades. This dichotomy is common to many famous computational problems; indeed, mathematical formulations of sequence assembly fall into a class of problems known as “NP-hard” that do not admit an easy solution (Medvedev et al. 2007).

There is another reason for continued advances in sequence assembly—advances in sequencing technology. As evident from the Staden quotes above, the first assembly methods were motivated by the invention of DNA sequencing and gel electrophoresis “readings” (Sanger and Coulson 1975; Maxam and Gilbert 1977). These early sequencing and assembly methods were applied to viruses with genomes of only a few kilobases (Sanger et al. 1978). As the sequencing technology was later commercialized and scaled, it became possible to assemble the 1-Mb genome of a free-living bacterium (Fleischmann et al. 1995), the 120-Mb genome of the fruit fly (Adams et al. 2000), and ultimately the 3-Gb human genome (Venter et al. 2001). These advances in scale required parallel computational advances, embodied by the tools that assembled these early genomes—TIGR Assembler (Sutton et al. 1995) and Celera Assembler (Myers et al. 2000). A similar theme continued through the 2000s, as new sequencing technology such as 454 (Margulies et al. 2005) and Illumina (formerly Solexa) (Bentley et al. 2008) required rethinking the assembly problem. The abrupt transition away from Sanger reads to the much shorter Illumina reads shifted the field toward de Bruijn graph assemblers (Pevzner et al. 2001) such as Velvet (Zerbino and Birney 2008) and ABySS (Simpson et al. 2009). Most recently, with the advent of longer, single-molecule sequencing reads from Pacific Biosciences (PacBio) (Eid et al. 2009), the field returned to overlap graphs (Myers 1995) and adaptations of Celera Assembler (Chin et al. 2013; Koren et al. 2013).

Presently, the number of available technologies has only grown. The papers in this issue include sequencing data from four platforms—Illumina, PacBio, Oxford Nanopore, and Sanger—and multiple technologies for constructing long-range scaffolds: paired reads, linked reads, optical mapping, proximity ligation, and physical mapping. This glut of technologies has spurred interest in determining the most effective approach to reconstruct whole genomes.

The low cost of short-read sequencing compared to Sanger has driven a wide expansion in the number of genomes sequenced, but with a sharp reduction in contig and scaffold lengths. An emerging trend is to combine cost-effective Illumina sequencing with clever library preparation techniques designed to improve assembly continuity. One powerful example is chromatin conformation capture via proximity ligation and high-throughput sequencing (Hi-C) (Lieberman-Aiden et al. 2009). This family of methods generates a familiar paired-read data type (two reads separated by some distance) but from a distribution of sizes that can span megabases. This data can be used to group contigs by chromosome, reconstruct chromosome-length scaffolds, and phase haplotypes (Burton et al. 2013; Kaplan and Dekker 2013; Selvaraj et al. 2013). In this issue, Rice et al. (2017) demonstrates a related approach, using in vitro reconstituted chromatin and Illumina sequencing to assemble the American alligator genome. Another approach to boosting short reads uses high-throughput barcoding to tag groups of “linked reads” that all originate from a larger, single molecule of DNA. For this new data type, Weisenfeld et al. (2017) introduces a new assembler, Supernova, for the de novo assembly of diploid human genomes from linked reads. Additionally, Jackman et al. (2017) describes a new version of the ABySS assembler and explores linked reads and optical mapping for improved scaffolding.

Although these short-read library preparation methods can extend scaffolds to span entire chromosomes, they lack the finer resolution required to improve contig lengths. Instead, the biggest gains in contig lengths have come from single-molecule sequencing. First from PacBio and most recently from Oxford Nanopore, these technologies can generate reads exceeding 10 kb, orders of magnitude longer than Illumina. Critically, 10-kb reads are longer than the most common repeats in both microbial and vertebrate genomes and can therefore generate highly continuous assemblies. In fact, the complete reconstruction of bacterial genomes—a process that used to require teams of people—is now automated and routine. However, the massive read lengths and increased error rate of these new technologies have also required updated assembly methods. This issue includes three new assembly tools designed specifically for long-read PacBio and Nanopore data: Canu (Koren et al. 2017), HINGE (Kamath et al. 2017), and Racon (Vaser et al. 2017).

Combining single-molecule sequencing with complementary technologies has also become a common strategy. Fan et al. (2017) demonstrates improved accuracy for human structural variant calling using a combination of PacBio and Illumina. For de novo assembly, two new studies look at plant genome assembly, with Zimin et al. (2017) combining PacBio and Illumina data to assemble the highly repetitive grass *Aegilops tauschii*, and Jiao et al. (2017) combining PacBio with proximity ligation and optical mapping to assemble relatives of the model species, *Arabadopsis thaliana*. In the latter case, the combination of PacBio reads and long-range scaffolding techniques enabled multi-megabase contigs and scaffolds spanning entire chromosome arms.

So, what is gained from improved de novo assemblies? High-quality assemblies can reveal repeat structures and structural variation otherwise missed by short-read resequencing. For de novo projects, short-read assemblies are fragmented, because the overlaps between reads are not long enough to distinguish between common repeats. These repeat sequences are left unassembled, breaking contigs and hindering analysis. With the emergence of long sequencing reads has come a renewed interest in repetitive sequences, which can be properly analyzed for the first time. This includes detailed analysis of highly repetitive satellite sequence in flies (Khosta et al. 2017) and birds (Weissensteiner et al. 2017), paving the way for functional studies in areas of the genome not previously accessible. Long-read assembly is even revealing new variation in the human genome, and Huddleston et al. (2017) highlights the importance of long-read sequencing and haplotype resolution for accurate structural variant detection.

Ultimately, the goal of genome assembly is a gapless, haplotype-resolved reconstruction, but these genomic jigsaw puzzles are so difficult that we have not yet finished the human genome. This issue marks the 38th build of the human reference sequence (Schneider et al. 2017), which still contains more than 800 gaps after decades of work and billions of dollars spent. But there is hope on the horizon. Progress over the past five years has been swift, driven by new technology. De novo assemblies of humans (Seo et al. 2016) and other vertebrates (Bickhart et al. 2017) are approaching reference quality by combining technologies such as PacBio, Illumina, optical mapping, linked reads, and proximity ligation; and new phasing methods can now recover chromosome-scale haplotype blocks from this data (Edge et al. 2017). With these latest techniques, only the largest segmental duplications and heterochromatic regions remain a challenge.

Future advances in technology may overcome these remaining hurdles. Reflecting on the earlier Staden quote, “If the overlap is of sufficient length to distinguish it from being a repeat …,” as soon as a sequencing technology can produce good enough reads, such that all regions of the genome can be uniquely distinguished, genome assembly will become trivial. Although the minimum combination of read accuracy and length required to complete the human genome is currently unknown, I suspect we are getting close. Most recently, Nanopore sequencing reads approaching 1 Mbp were reported (http://lab.loman.net/2017/03/09/ultrareads-for-nanopore/), and it is imaginable that further technology advances will enable the complete assembly of a diploid human within a few years.

Luckily, even if sequence assembly is made obsolete by new technology, there will be plenty of work left for the bioinformaticians. Low-cost, complete genomes will enable new and powerful comparative genomics studies, requiring scalable methods for analyzing whole genomes. Many of these methods share much in common with the de Bruijn and overlap graphs of genome assembly. Illustrating these connections, Paten et al. (2017) provides a state-of-the-art overview on the topic of genome graphs and their application to read mapping, variant calling, and haplotype determination. These graph structures allow reference genomes to evolve beyond a single, linear representation to capture the full diversity of a population. Hopefully, as continued advances in technology allow us to spend less time assembling genomes, we can spend more time exploring their wonderful evolution and functional complexity.

## Competing interest statement

A.M.P. served as a guest editor for this issue of *Genome Research*, had access to all papers prior to publication, and coauthored two included papers.
